# Pattern of bacterial infection in liver transplantation

**DOI:** 10.1186/1753-6561-5-S6-P50

**Published:** 2011-06-29

**Authors:** N Khamis, I Kamel, G Fahmy, S Mokhtar, A Tohamy

**Affiliations:** 1 Infection Control, Ain Shams University Specialized Hospital, Cairo, Egypt; 2 ASCOT, Ain Shams University Specialized Hospital, Cairo, Egypt

## Introduction / objectives

Bacterial infection frequently occurs early after liver transplantation. It is recognized as important complication which might interfere with the outcome of such life saving operations.

Various studies have characterized the pathogens, times of onset and sites of infection, however risk factors interacting together to end with bacterial infections have not fully defined and important issues remain unsettled.

## Methods

In Ain Shams University Specialized Hospital (ASUSH), a descriptive epidemiologic study was conducted over two year’s period,from March 2008 through February 2010.The study included 64 patients divided equally into 32 donors and 32 recipients The infections were in different sites and the most predominant microorganisms were the gram-negative. Investigation of probable leading factors for post operative infections was done and an intervention protocol was formulated and implemented. It included preoperative bacterial screening of recipient and a bundle of measures for SSI prevention.

## Results

The total infection rates in recipients was 40.6% and in donors 18.75%.The most common type of infection was the surgical drain followed by the bile drain, which are also risk factors for infections. Infections with gram negative bacteria were more prevalent (66.6%) with predominance of Pseudomonas spp (37.5%).The rate of Gram positive bacteria was 33.3% and MRS was the predominant type of bacteria (%53.57%).

## Conclusion

The high incidence of gram negative bacterial infections encountered in the actual study could be referred to the underlined clinical condition of cirrhosis on top of hepatitis “C” (HCV) chronic infections; which favors the colonization of this type of bacteria in abdominal lymphatic. A bundle of SSI prevention will be of value for those patients.

## Disclosure of interest

None declared.

